# Pelvic venous congestion syndrome: female venous congestive syndromes and endovascular treatment options

**DOI:** 10.1186/s42155-023-00365-y

**Published:** 2023-04-20

**Authors:** Elika Kashef, Elizabeth Evans, Neeral Patel, Deepsha Agrawal, Anne P Hemingway

**Affiliations:** 1 Department of Radiology, Imperial College Healthcare NHS Trust, The Bays, South Wharf Road, London, W2 1NY UK; 2 Imperial College Healthcare NHS Trust, St Mary’s Hospital, S Wharf Road, London, W2PE UK; 3 Greater Glasgow and Clyde, Queen Elizabeth University Hospital, Glasgow, G12 0XH UK; 4 Department of Radiology, Oxford University Hospital NHS Foundation Trust, Oxford, OX39DU ST3 UK

**Keywords:** Chronic pelvic pain, Pelvic congestion syndrome, Pelvic venous insufficiency, Ovarian varices, Embolization, Embolotherapy, Pelvic varices, Ovarian Vein Embolization

## Abstract

Pelvic venous congestion syndrome (PVCS) is a common, but underdiagnosed, cause of chronic pelvic pain (CPP) in women.

PVCS occurs usually, but not exclusively, in multiparous women. It is characterized by chronic pelvic pain of more than six months duration with no evidence of inflammatory disease.

The patients present to general practitioners, gynaecologists, vascular specialists, pain specialists, gastroenterologists and psychiatrists. Pain of variable intensity occurs at any time but is worse in the pre-menstrual period, and is exacerbated by walking, standing, and fatigue. Post coital ache, dysmenorrhea, dyspareunia, bladder irritability and rectal discomfort are also common. Under-diagnosis of this condition can lead to anxiety and depression.

A multidisciplinary approach in the investigation and management of these women is vital.

Non-invasive imaging (US, CT, MRI) are essential in the diagnosis and exclusion of other conditions that cause CPP as well in the definitive diagnosis of PVCS. Trans-catheter venography remains the gold standard modality for the definitive diagnosis and is undertaken as an immediate precursor to ovarian vein embolization (OVE). Conservative, medical and surgical management strategies have been reported but have been superseded by OVE, which has a reported technical success rates of 96–100%, low complication rates and long-term symptomatic relief in between 70–90% of cases.

The condition, described in this paper as PVCS, is referred to by a wide variety of other terms in the literature, a cause of confusion.

There is a significant body of literature describing the syndrome and the excellent outcomes following OVE however the lack of prospective, multicentre randomized controlled trials for both investigation and management of PVCS is a significant barrier to the complete acceptance of both the existence, investigation and management of the condition.

## Background

Chronic pelvic pain (CPP) is defined by the American College of Obstetricians and Gynaecologists as ‘pain symptoms perceived to originate from the pelvic organs typically lasting more than 6 months. It is often associated with negative cognitive, behavioural, sexual and emotional consequences as well as with symptoms suggestive of lower urinary tract, sexual, bowel, pelvic floor, myofascial or gynaecological dysfunction’ (Pain 2020).

CPP is estimated to affect 10 million women worldwide, up to 7 million of whom do not seek medical assistance (Perry 2001).It is thought to account for up to 10–20% of all gynaecology outpatient appointments, and is an indication for 20–33% of all diagnostic laparoscopies. CPP has a population prevalence of 15% in women aged 18–50 years, in 61% of cases the aetiology of the pain is unexplained (Perry 2001; Ignacio 2008; Phillips et al. 2014; Gynaecological 1978; Mathias et al. 1996).

Causes of CPP include endometriosis, adenomyosis, fibroids, musculoskeletal disorders, chronic pelvic inflammatory disease, irritable bowel syndrome, painful bladder syndrome, pelvic venous congestion syndrome and psychological factors.

In 1857 French anatomist and surgeon, Louis Alfred Richet was the first to observe an association between chronic pelvic pain and the presence of varicose veins in the utero-ovarian plexus (Richet 1857). The important association of CPP, broad ligament varicocele and multiparity was made by Lefevre (Lefevre 1964). The demonstration of retrograde filling of the left ovarian vein during renal venography led specialists to consider pelvic varicosities to be analogous to scrotal varicoceles in men (Chidekel 1968).

The presence of lower extremity and vulval varices associated with varices in the pudendal, peri-labial, gluteal and posterolateral thigh regions in women referred with CPP/PVCS is recognised to be important for diagnosis and to determine the most appropriate management strategy (Hobbs 1976).

Different terms have been used interchangeably to describe the entity which these authors refer to as the Pelvic Venous Congestion Syndrome (PVCS). Characterized by chronic pelvic pain and para-metrial tenderness caused by dilatation of the ovarian and/or pelvic veins, PVCS has been described as the chronic pelvic pain syndrome (CPPS), pelvic congestion syndrome (PCS), female varicocele and pelvic venous congestion (PVC). Pelvic vascular insufficiency (PVI) is used to describe patients where the aetiology of PVCS is incompetent or absent gonadal vein valves.

The varied terminology and continued dispute regarding the existence of pelvic venous disorders (PeVDs) has consequences for methods of diagnosis and treatment (Khilnani 2019; Campbell et al. 2020). In an attempt to resolve this controversy, Meissner et al. published The Symptoms-Varices-Pathophysiology (SVP) classification of pelvic venous disorders (Meissner et al. 2021). This classification, defines three domains Symptoms (S), Varices (V) and Pathophysiology (P). This latter domain encompasses anatomy (A), hemodynamic (H) and aetiologic (E) features, so that an individual’s classification is designated SVP_AHE._ In this classification PeVDs occur in four anatomic zones. The 4^th^zone is classified within the internationally recognised CEAP (clinical, etiologic, anatomic physiologic) system which exists for classifying lower extremity venous disorders. This system improves communication diagnosis, management and research (Lurie et al. 2020)although it’s utility in the clinical setting and in determining treatment has yet to be determined. The reader is referred to the excellent graphical abstracts for both systems (Meissner et al. 2021; Lurie et al. 2020) Fig.1.

**Fig. 1:**
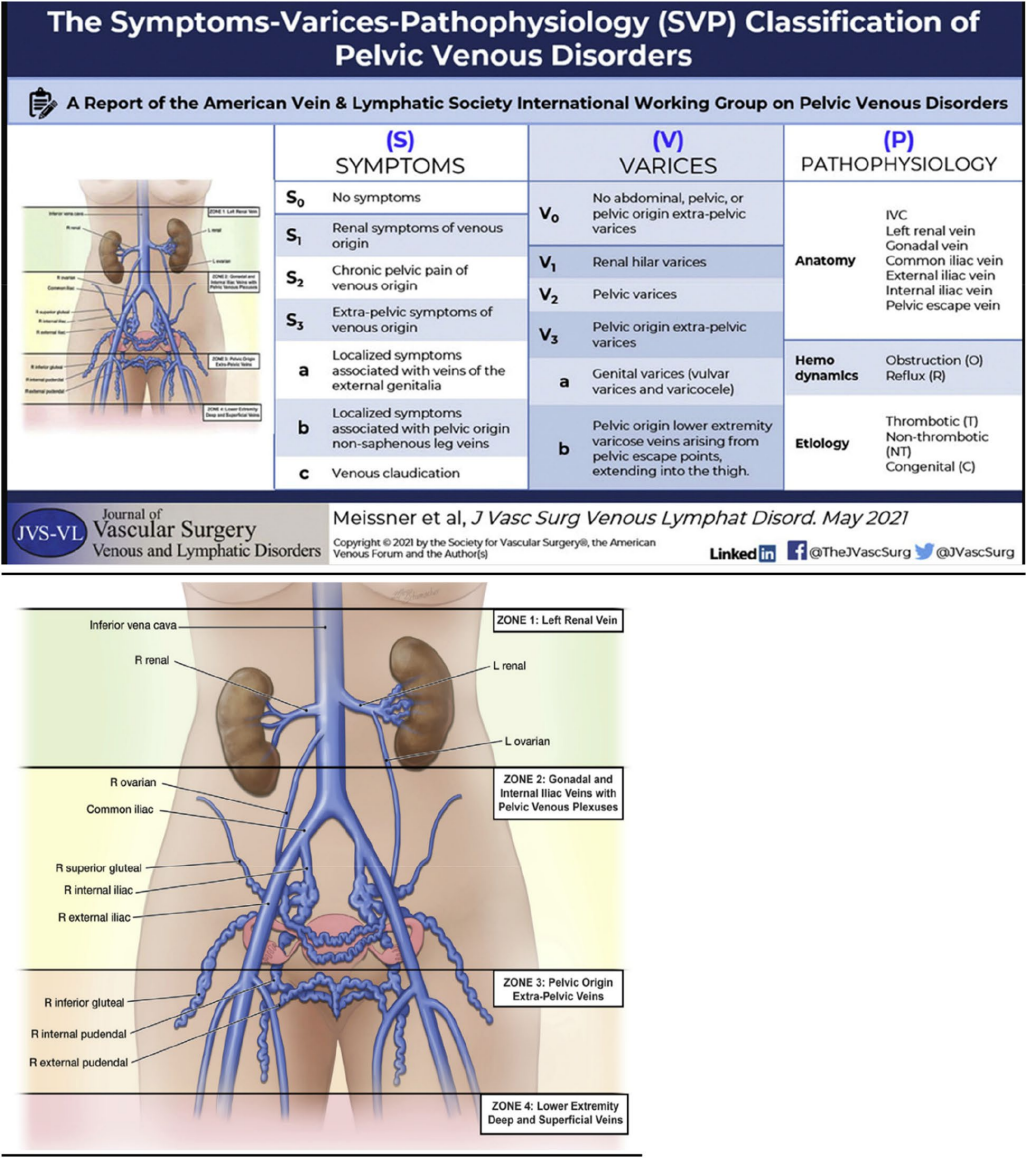
Symptoms-Varices-Pathophysiology (SVP) classification (Meissner et al 2021) Reproduced under Creative Commons Licence. Images Acknowledged to Mesa Schumacher 2021)

Constitutional, mechanical, inflammatory, hormonal, neural, psychosomatic and vascular aetiologies have all been postulated for PVCS (Hobbs 1976). Anxiety and depression in this group of women has long been recognized with much debate as to whether this cause or effect (Beard et al. 1986). PVCS occurs as a result of primary intrinsic venous abnormalities such as absence of valves and venous incompetence, and secondary to mechanical factors causing venous obstruction including Nutcracker and May-Thurner syndromes.

## Anatomy

**Fig. 2:**
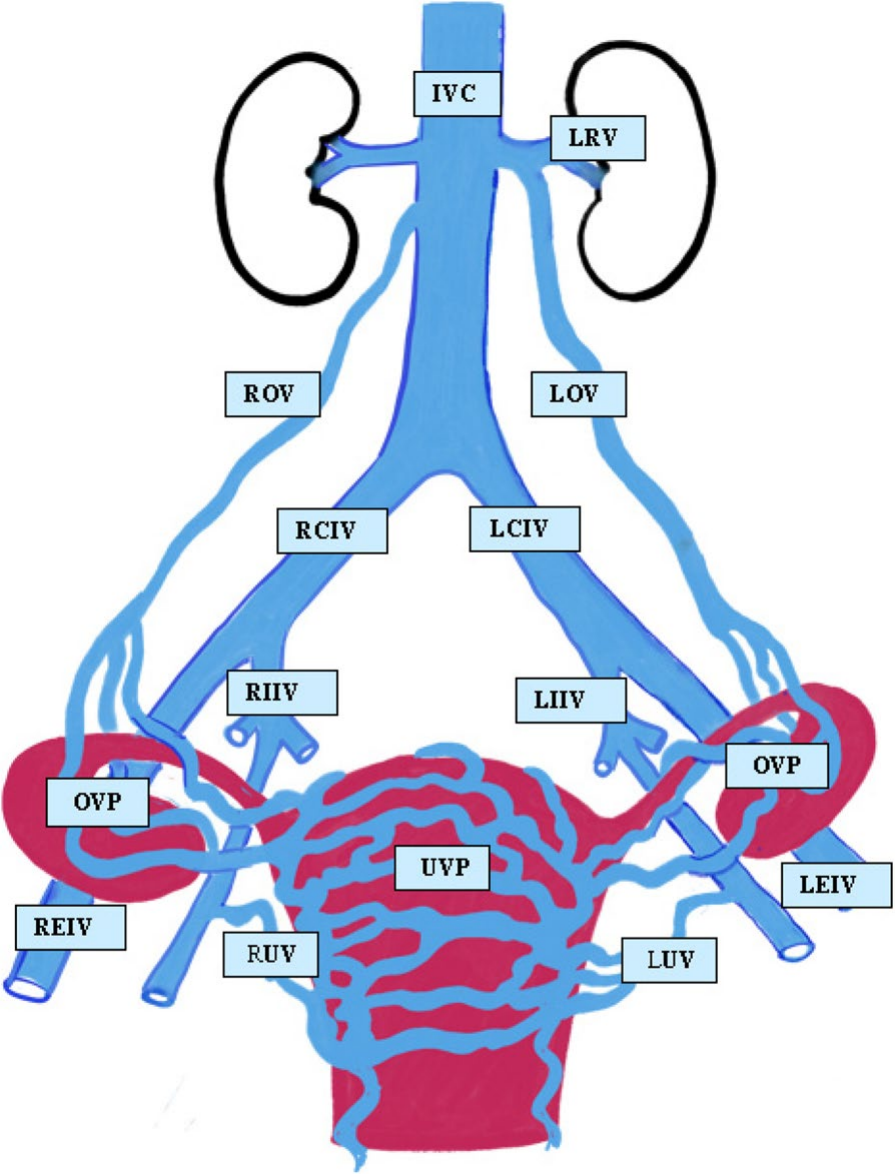
Normal pelvic and ovarian venous anatomy. The rich uterine venous plexus (UVP) drains via the right and left uterine veins (RUV, LUV) into the internal iliac veins (RIIV, LIIV) which anastomose with the external iliac veins (EIV) to become the common iliac veins (CIV). The UVP anastomoses superiorly with the ovarian venous plexus (OVP) bilaterally which drain into the ovarian veins. The right ovarian vein (ROV) drains into the inferior vena cava (IVC) and the left ovarian vein (LOV) drains into the left renal (LRV). (Image courtesy S Boland)

The venous drainage system within the pelvis is rich, variable and complex (Venbrux 2012). The major vessels draining female pelvic viscera are the ovarian veins and the common, external and internal iliac veins, the latter having parietal and visceral branches (Kennedy and Hemingway 1990) (Fig.2).

The ovarian veins form a rich venous-anastomotic plexus bilaterally in communication with the plexus draining the broad ligament and fallopian tube as well as the uterine fundal venous plexus. Ovarian veins are formed by the union of two or three tributaries that meet at the level of the fourth lumbar vertebra. The left ovarian vein (LOV) almost invariably drains into the left renal vein, while the right ovarian vein (ROV) drains into the inferior vena cava although it may drain into the right renal vein in up to 10% of cases (Freedman et al. 2010). On the left, there is a recognized although uncommon communication between the LOV and inferior mesenteric vein. Valves may be absent from the cranial portion of the LOV in 13–15% of women and from 6% in the ROV (Ahlberg et al. 1966). Valves may incompetent in 43% on the left and 35–41% on the right.

Intercommunicating plexuses from visceral branches of the internal iliac vein include:

- Vaginal plexus and uterine plexus draining via the uterine veins, usually three on each side running laterally to drain into the internal iliac vein (IIV). The fundal uterine plexus drains into both the uterine veins and ovarian venous plexus
- Vesical plexus
- Deep clitoral, labial and inferior rectal veins drain into the internal pudendal and inferior gluteal veins
- Rectal branches communicate with the utero-vaginal plexus

The parietal branches of the IIV include the superior and inferior gluteal veins, iliolumbar veins, and the sacral venous plexus and obturator veins which may drain into the external iliac venous system. The pelvic venous system has numerous potential collateral pathways, the reader is referred to a comprehensive pictorial review of these by Zurcher et al. (Zurcher 2022).

The Nutcracker or left renal vein entrapment syndrome refers to compression of the left renal vein between the aorta and superior mesenteric artery. This can result in increased pressure in the left renal vein with reflux into the ovarian vein possibly leading to pelvic varices (Coakley et al. 1999).

May-Thurner syndrome refers to chronic compression of the left iliac vein against the lumbar spine by the overlying right common iliac artery. It can result in chronic deep venous thrombosis which can ultimately divert blood to the left internal lilac vein and give rise to pelvic varices.

## Management

Management of PVCS including diagnosis, investigation and treatment requires a multidisciplinary team approach involving gynaecologists, vascular surgeons, diagnostic and interventional radiologists, urologists, neurologists, psychologists and psychiatrists (Ignacio 2008; Cordts et al. 1998; Osman et al. 2021; Drife 1993).

### Diagnosis

Diagnosis of PVSC /PVI is based on the clinical history, presentation, and physical examination and imaging investigations. Patients are usually, although not exclusively, pre-menopausal and multiparous. PVCS is characterized by chronic pelvic pain of more than six months duration with no evidence of inflammatory disease. Pain of variable intensity may be uni- or bilateral but is usually asymmetric, is worse premenstrually, exacerbated by walking, standing, and fatigue. Post coital ache (65%), dysmenorrhea (66%) and dyspareunia (71%), bladder irritability and rectal discomfort are common (Taylor 1949; Beard 1988; Kuligowska et al. 2005). Under-diagnosis can lead to anxiety and depression (Hobbs 1976). Beard et al. observed that post-coital ache and/or ovarian point tenderness, occurring in 86% of their series, were strong discriminators in favour of PVCS demonstrating 94% sensitivity and 77% specificity (Beard et al. 1986; Beard 1988).

Not all women with PVCS have typical pelvic pain, they may present with recurrent lower extremity varicose veins or hip pain (Phillips et al. 2014). A case of persistent genital arousal, an under-recognised and distressing condition, was found to have PVCS which was successfully treated with OVE (Thorne and Stuckey 2008).

### Investigations

Investigations include laparoscopy, non-invasive and invasive imaging techniques.

#### Laparoscopy

Laparoscopy is commonly undertaken in the investigation of women presenting with CPP. Beard et al. found dilated veins and vascular congestion in the broad ligament and ovarian plexus in 91% of women examined laparoscopically for CPP with no other pelvic pathology (Beard et al. 1984). Whilst laparoscopy is excellent for identifying other pelvic pathology that may cause CPP, because the patient is supine and CO_2_is insufflated, varices may be compressed and the diagnosis of PVCS masked in as many as 86–90% of cases (Ignacio 2008).

#### Imaging

PVCS can exist in isolation or combination with other causes of CPP. Imaging should be used to exclude other causes of CPP as described above, as well as confirm a clinically suspected diagnosis of PVCS (Bookwalter et al. 2019). Transabdominal (TA) and transvaginal (TV) ultrasound, magnetic resonance imaging (MRI), magnetic resonance venography (MRV), computed tomography (CT) and venography are all utilized in the investigation of CPP/PVCS.

#### Non-invasive imaging

##### Ultrasound

TAU and TVU should be combined with colour Doppler imaging (CDI) and Doppler spectral analysis (Kuligowska et al. 2005; Stones et al. 1990; Hodgson et al. 1991; Lemasle and Greiner 2017).

**Fig. 3:**
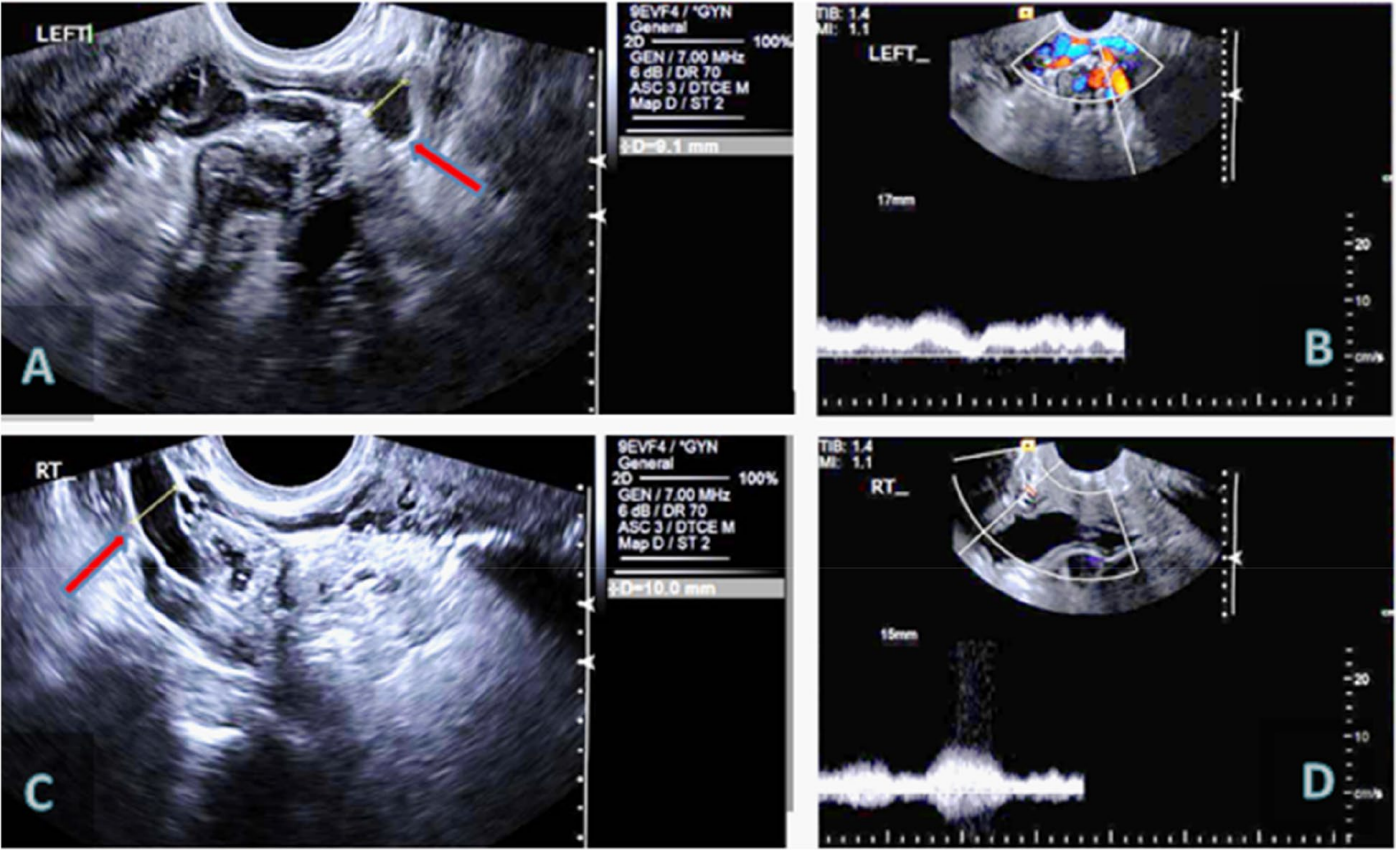
Trans-vaginal and Doppler ultrasound studies in a multiparous woman presenting with bilateral lower limb varicosities and pelvic pain. Pelvic venous congestion with left ovarian vein diameter of 9.1mm (**A**), and right ovarian vein diameter of 10mm (**C**) (red arrows), both diameters increased with Valsalva manoeuvre. Left (**B**) and right (**D**) ovarian vein Doppler studies Average flow 0.8cm/sec

Park et al. examined 32 women with PVCS and 35 control subjects (Park et al. 2004). A dilated left ovarian vein with diameter of 6 mm or greater with reversed caudal flow gave a positive predictive value of 83.3%. They described dilated, tortuous pelvic venous plexuses, polycystic ovarian changes and dilated arcuate veins, greater than 5 mm in diameter, crossing the uterine myometrium between pelvic varicosities and a variable duplex waveform during provocation with a Valsalva manoeuvre and slow venous flow of less than 3 cm/s (Fig. 3).

Polycystic ovaries have been reported in up to 40% of women with pelvic venous insufficiency and venous congestion, an association which may be related to hormonal factors.(Bookwalter et al. 2019; Park et al. 2004).

Diagnostic criteria for the diagnosis of PVCS on ultrasound are (Park et al. 2004):

- Tortuous pelvic veins diameter > 6 mm
- Slow blood flow < 3 cm/sec or reversed caudal flow
- Dilated arcuate veins in the myometrium communicating between bilateral pelvic varicose veins
- Polycystic changes in the ovaries

##### Computed tomography

Abdominal and pelvic computed tomography (CT) is commonly undertaken in the evaluation of women with CPP. A study of the CT and MR appearances of pelvic varices, characterized their appearances as dilated tortuous para uterine tubular structures extending laterally in the broad ligament and reaching the pelvic side wall or extending inferiorly to communicate with the paravaginal venous plexus (Coakley et al. 1999).

The following criteria were suggested for diagnosis of pelvic varices: at least 4 ipsilateral tortuous para-uterine veins of varying calibre at least one with a diameter of > 4 mm or an ovarian vein diameter of > 8 mm (Coakley et al. 1999). CT may be of specific value in identifying the Nutcracker and May-Thurner syndromes (Osman et al. 2021; Szaflarski et al. 2019) but MR should remain the first line of investigation in this cohort due the patient young age and radiation dose.

##### Magnetic resonance imaging

MRI is non-invasive, does not utilize ionizing radiation and provides excellent imaging of the many causes of CPP (Coakley et al. 1999; Kuligowska et al. 2005).

**Fig. 4:**
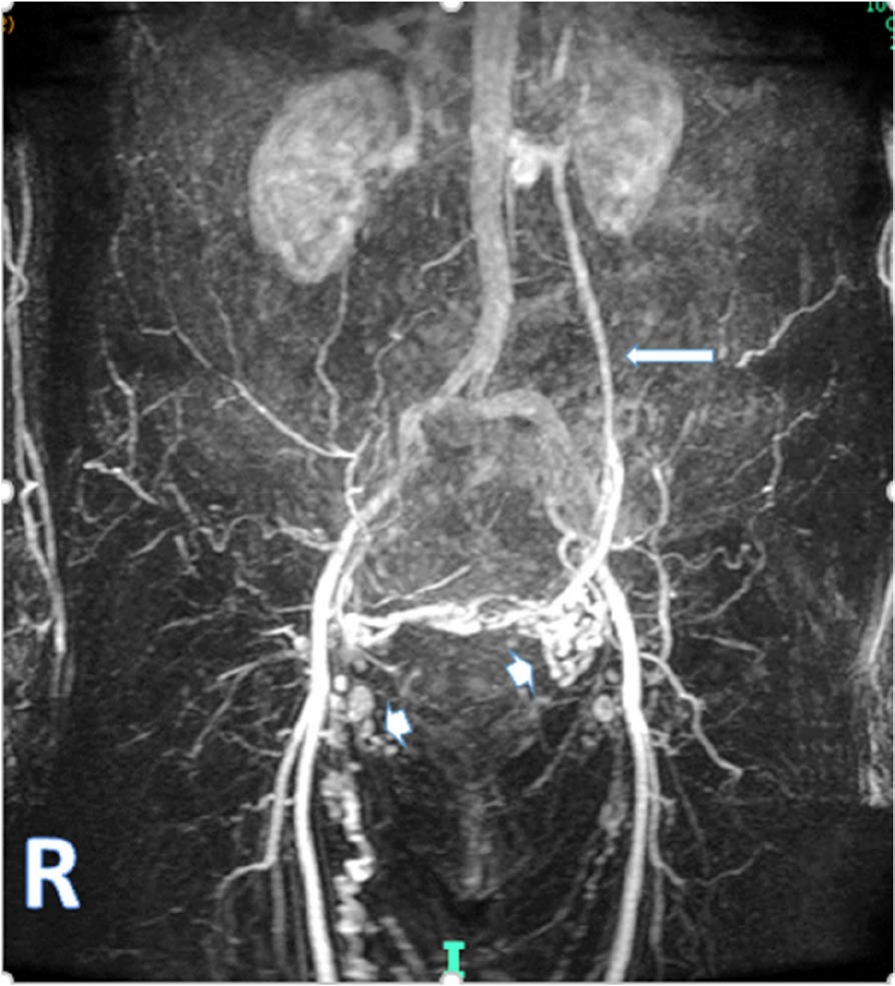
MRI/MRv The left ovarian vein (long arrow) is clearly identified filling early and extends down into the pelvis where small varices (arrow head) are filled on the left side with a crossover of venous flow to the right side and subsequent multiple varicosities are identified in the right upper thigh on this TR IC K S study

A prospective study has shown that MRV shows concordance with phlebography in 96% of cases for venous anatomy and 70% for grade of venous congestion with a sensitivity and specificity of 88% and 67% for ovarian veins, 100% and 38% for internal iliac veins and 91% and 42% for the pelvic plexus (Asciutto et al. 2008). Time resolved MR angiography has been reported to be of value in detecting gonadal vein dilatation and reflux (Dick et al. 2010) (Fig. 4). In a comparison of time resolved MR angiography with venography the specificity, sensitivity and accuracy were assessed respectively as 61–75%, 100% and 79–84% (Yang et al. 2012). Velocity-encoded gradient-echo MRV when compared with catheter venography is reported to have a sensitivity of 88% and specificity of 67% (Barge 2022).

CT and MRI both performed with the patient supine may underestimate the extent of venous dilatation that is demonstrated by ultrasound and venography where provocation with the Valsalva manoeuvre can be employed.

##### Venography

Catheter venography remains the gold standard for the diagnosis for PVCS and PVI and is undertaken when non-invasive studies are inconclusive in the presence of a strong clinical history and as an immediate precursor to trans-catheter management of pelvic varices (Kennedy and Hemingway 1990; Freedman et al. 2010).

Prior to the more routine use of catheter venography trans-uterine venography and direct vulval venography were performed.

##### Trans-Uterine venography

This procedure, no longer used in common practice, involved the direct injection of contrast medium into the fundal myometrium (Guilhem 1954; Kauppila et al. 1971; Silverberg et al. 1973). The procedure was reported as being well tolerated by the women examined (Kennedy and Hemingway 1990; Beard et al. 1984). A scoring system was utilized to determine if the venographic appearances were commensurate with the diagnosis of pelvic congestion. A value of 5 or more gave a diagnostic sensitivity of 91% and specificity of 89% for PPS (Beard 1988).

##### Vulval venography

The direct cannulation of vulval varices in women with symptoms suggestive of PVCS has been described (Craig and Hobbs 1975; Thomas et al. 1967). Communication with the external pudendal, femoral and saphenous veins, and in severe cases communication with vesical venous plexus, broad ligament plexus and internal iliac vein was demonstrated.

**Fig. 5:**
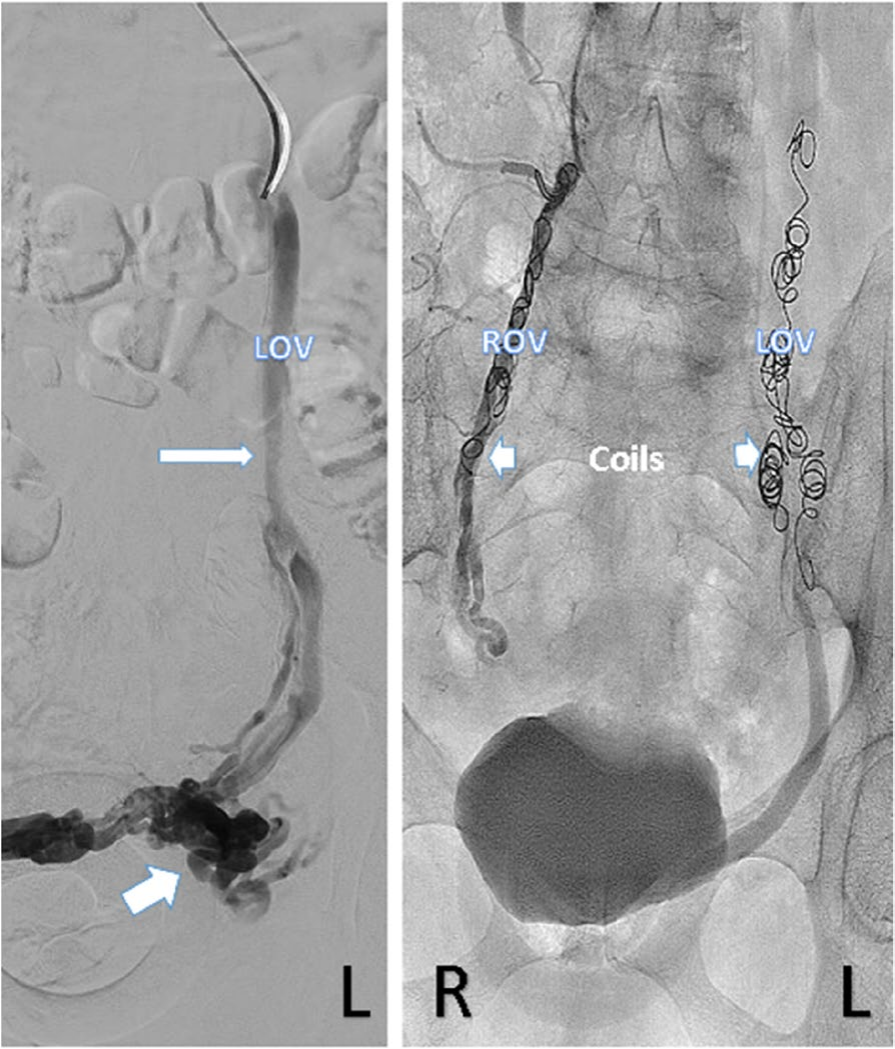
Left ovarian venogram demonstrating left ovarian vein (LOV) dilatation with dilated pelvic and para uterine veins. The left ovarian vein was treated with 3% STS and coil embolization. The right ovarian vein (ROV) did not appear dilated and was treated with coil embolization

The technique, for diagnostic purposes, has been replaced by trans-catheter venography (Fig. 5) however direct puncture sclerotherapy of vulval varices may be utilized as an adjunct treatment in the management of PVCS.

## Treatment options

Multiple treatment approaches have been studied and used for the treatment of PVCS and include conservative, psychological, medical, surgical and endovascular options either in isolation or combination.

Chronic debilitating pelvic pain is often accompanied by depression and anxiety. An RCT comparing medroxyprogesterone acetate (MPA) and psychotherapy found that 9 months after treatment was ended 72% of women treated with both showed a ≥ 50% reduction in pain score, superior to either treatment being used in isolation (Farquhar 1989).

Medical therapy includes analgesia, non-steroidal anti-inflammatory agents (NSAIDs), gonadotropin-releasing hormone (GnRH) antagonists with hormone replacement therapy (HRT), dihydroergotamine, progestins, medroxyprogesterone acetate (MPA) and Goserelin acetate (Ignacio 2008; Venbrux 2012). Long term pharmacological therapy is not recommended for treatment of PVCS because of adverse symptoms and limited efficacy (Knuttinen et al. 2015).

Surgical treatment options include laparoscopic ovarian vein ligation, abdominal hysterectomy and oophorectomy. Gargiulo et al. reported 100% symptom remission in 23 women 12 months following laparoscopic trans-peritoneal ovarian vein ligation (Gargiulo et al. 2003).

In a study in which women with PVCS who had not responded to medication were randomised to either embolization, or hysterectomy and bilateral oophorectomy with HRT or hysterectomy with unilateral oophorectomy embolization was found to be significantly more effective (*p *< 0.05) at reducing pain than the other two methods (Chung and Huh 2003).

Hysterectomy may be offered when all other treatments have failed but may not be curative, 22–33% of women may continue to suffer pain (Drife 1993; Stovall et al. 1990; Beard 1991).

## Endovascular management

OVE is recommended by the Society of Vascular Surgery with a 2B level of evidence: ‘We suggest treatment of pelvic congestion syndrome and pelvic varices with coil embolization, plugs, or trans-catheter sclerotherapy, used alone or together’ (Gloviczki 2011).

### Transcatheter embolization

Following the initial report in 1993 of ovarian vein embolization (OVE) for the treatment of a patient with CPP/PPS secondary to PVCS the technique has evolved to become the mainstay of treatment for this condition (Edwards et al. 1993). Embolization is preceded by selective ovarian and iliac venography to delineate the anatomy and identify all relevant vessels and collateral pathways (Kennedy and Hemingway 1990; Jacobs 1969). Contraindications include active pelvic infection, severe contrast medium allergy, coagulopathy and pregnancy.

Patients assessed as suitable for consideration for OVE by the MDT must undergo pre-procedure consultation. At our institute, this occurs alongside the TV ultrasound assessment. A patient information leaflet is provided and procedural risks together with the rate of recurrence, failure to improve symptoms and coil migration are explained. A second consultation is arranged to ensure the patient has considered the options and wishes to proceed.

Patients are required to be nil by mouth. It is a requirement that someone to accompany them home and stays with them for 24 h following sedation (sedo-analgesia). A proposed patient pathway is described in Table 1.

**Table 1:**
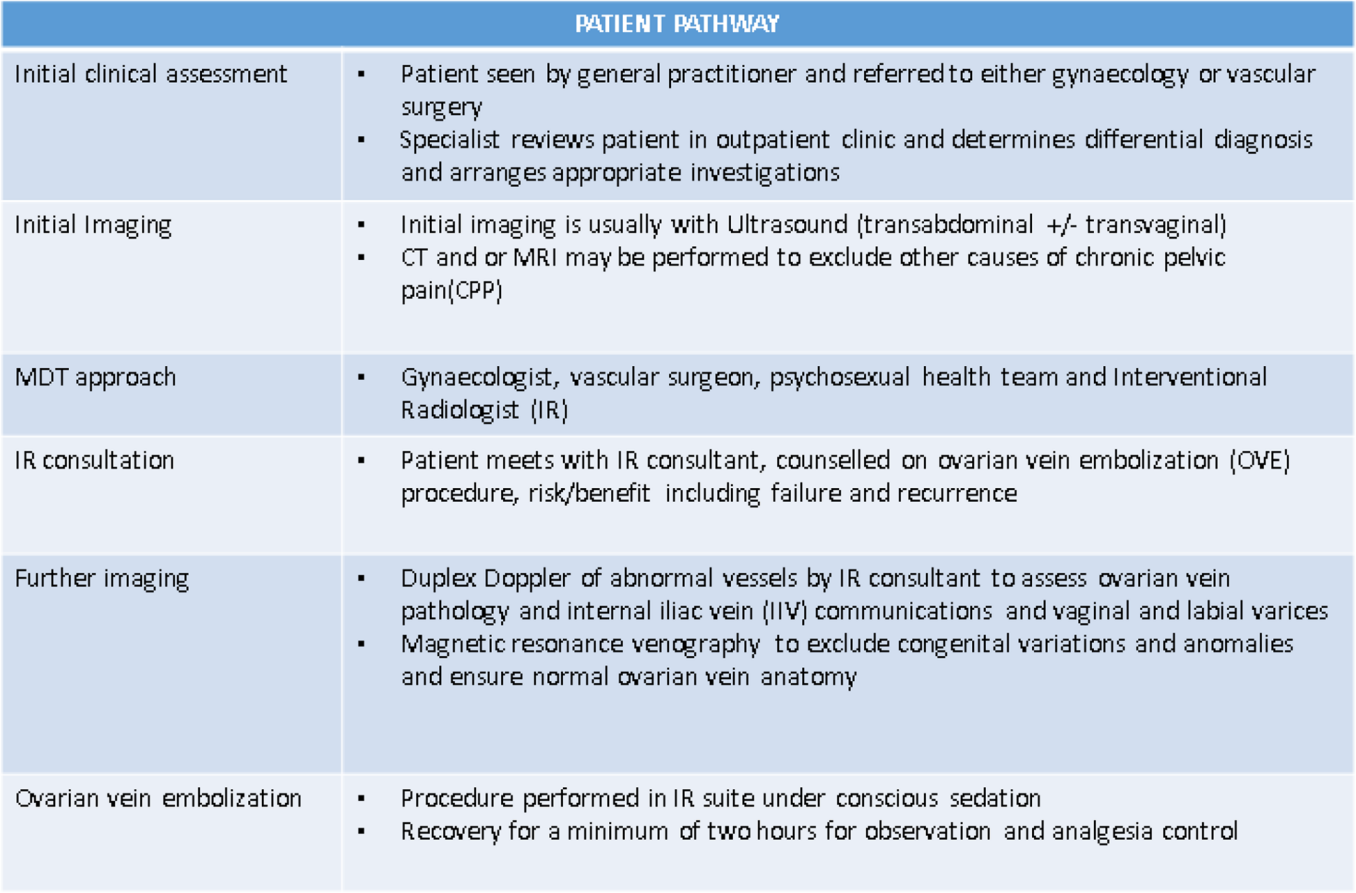
Provides a suggested patient pathway for the management of patients with PVCS

#### Technique (Venbrux 2002; Lopez 2015)

The patient is admitted to the IR Day-case facility and informed consent obtained. Pregnancy should be excluded in women of childbearing age.

The WHO safety checklist is utilized and IV conscious sedation (e.g. fentanyl and midazolam) is readily available (WHO). The patient’s vital signs are monitored.

The procedure is undertaken under sterile conditions employing local anaesthesia for venous access, under ultrasound guidance. Right internal jugular vein (IJV) or the common femoral vein (CFV) are both suitable access options. There is a preference for the RIJV approach as this offers a ‘downhill’ approach to the ovarian and pelvic veins. Other access points such as brachial and subclavian veins have also been used but have not shown to be superior to the IJV or CFV approach (Lopez 2015).

Selective catheterization of the left and right ovarian veins is undertaken with and without provocation such as Valsalva manoeuvre. Ovarian vein cannulation can be achieved with a 5F Multipurpose or a shaped tip catheter such as a Bernstein (Merit Medical, Utah, US) with an 0.035″or 0.038″ lumen based on the type of the coil to be utilised. Some detachable coils will require use of a microcatheter on a 0.018″ platform.

Distal vessel embolization with a sclerosant such as 3% sodium tetradecyl sulphate (STS) can be prepared as a foam using the Tessari method (± balloon occlusion to aid stagnation) or injected as part of the sandwich technique where coil and foam are used in combination (Tessari et al. 2001; Xu et al. 2016).

The maximum dose of 3% STS recommended per procedure is 10 ml. STS is often injected in foam state mixed with room air, O2 or CO2. The higher concentration of nitrogen in room air poses a potential risk of air embolus and stroke when in Trendelenburg position (Tessari et al. 2001).

The radiolucent property of STS foam can be overcome by filling the dead space of the catheter with contrast and pulsed controlled injection under fluoroscopic guidance. Injecting foam distally can ensure smaller pelvic branches are effectively embolized. This technique followed by coil embolization has offered the best outcomes for patients.

The entire length of the refluxing, incompetent vessel is embolized with MR compatible platinum coils to prevent collateralisation. It is not necessary to tightly pack the coils within the ovarian vein, but sufficient should be deployed to slow the flow, induce thrombosis and block any tributaries. Under-coiling of the ovarian veins can increase probability of recurrence. Oversizing of coils is important to prevent migration. Coil size depends on ovarian vein diameter. Common sizes range 8 mm-20 mm with longer lengths used to cover the entire vein. Distal embolization is with push-able coils however for the more proximal deployment, detachable coils allow for more accurate placement as well as “test” deployment to ensure no migration risk.

Examination of the internal iliac veins is also undertaken and embolization using a balloon occlusion technique, sclerosant and coils if necessary can be carried out at the same procedure or a staged procedure some weeks later. Foam sclerotherapy of pudendal and broad ligament branches (± coil embolization) is effective. Coil embolization below the inguinal ligament is not recommended as this can be palpable and cause discomfort long-term.

Other embolic agents used in isolation or in combination include:

- Sclerotherapy products e.g. 5% morrhuate of sodium, sodium tetradecyl sulphate, (STS), n-butyl-2-cyanoacrylate (glue), Ethylene vinyl alcohol copolymer and lauromacrogol 400.
- Occlusive detachable plugs
- Embolization coils – platinum coils (MRI compatible to 1.5 Tesla) – Pushable or detachable. Nickel free coils are available for those with allergy to nickel.

**Fig. 6:**
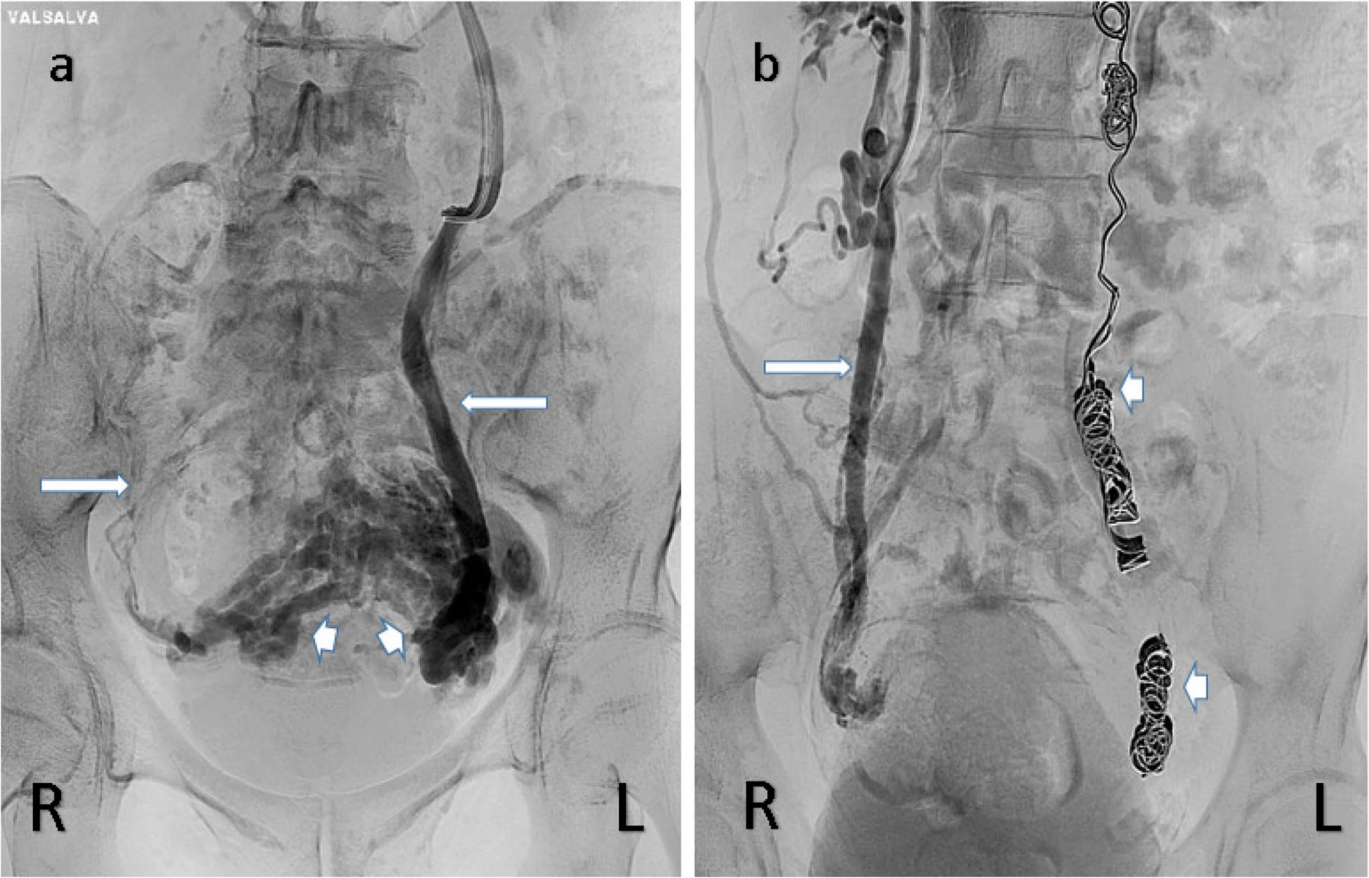
6**a** Left Ovarian venography with Valsalva The left ovarian venogram (long arrow on left) in a multiparous woman, shows significant venous dilatation with extensive varicosities (arrow heads) and reflux across the midline to the right ovarian vein (long arrow on right). 6**b** Right ovarian venography following left ovarian vein coil embolization (arrow heads). The ROV (long arrow) was embolized with coils

Venography after embolization of the ovarian veins is undertaken to confirm vessel occlusion (Figs. 6).

Manual compression of the RIJ or other venous access route following catheter removal is followed by 2 hours bed rest post procedure. The patient is rested in the semi-recumbent position at 45 degrees following an IJV puncture.

If vulvar varices do not resolve or improve consideration can be given to direct percutaneous STS foam sclerotherapy; this should be used as an adjunct to the treatment of reflux not as a stand-alone treatment in isolation (Bookwalter et al. 2019).

Nasser reported a marked association between lower extremity varicose veins (VVs) and PVCS of 92.9% in his series and lower rates of VV recurrence in patients who underwent VV treatment after OVE for PVCS (Nasser et al. 2014; Laborda et al. 2013; Meneses et al. 2013).

May-Thurner syndrome (MTS), is an uncommon condition that may be found in association with PVCS. Stenting of the common iliac vein (CIV) can provide significant amelioration of symptoms. Ahmed reported a technical success rate of 100% (34 of 34) (Ahmed et al. 2016). No major complications occurred, and 68% of patients (23 of 34) had clinical success with relief of presenting symptoms on follow-up visits. Gavrilov et al. looked at endovascular interventions in the treatment of PVCS caused by MTS (Gavrilov et al. 2020). In a small series stenting CIV was effective in only 16.6% of patients but when combined with embolization of the ovarian veins 83.4% of patients experienced symptom elimination.

The role of embolization of left ovarian vein in presence of Nutcracker syndrome remains controversial with no definite consensus and is beyond the scope of this paper.

### Complications

Procedural complications are unusual with overall rates reported as between 2%-3% (Venbrux 2012; Freedman et al. 2010; Venbrux 2002). Infection risk is thought to be approximately 1%. Coil migration is reported especially when the internal iliac vein branches are embolized. Coils may migrate to the right ventricle and pulmonary circulation with the potential to cause acute or chronic complications such as arrhythmias and thrombosis (Scott and Cullen 2021). Coils should be snared and retrieved at the time of the procedure. Contrast allergy is rare (< 1%). Puncture related hematoma, thrombophlebitis, vessel perforation, non-target embolization and pneumothorax have been reported.

Post procedural discomfort and pain are managed with appropriate analgesia. Use of NSAIDs may alleviate some of the local discomfort which arises from phlebitis.

Delayed complications include recurrence in 10–40% of cases (Meneses et al. 2013). Recurrence is higher in patient with untreated lower extremity varicosities.

Many of the women undergoing OVE are of childbearing age. Machine parameter should be optimized, tight collimation and experienced operators help to keep the radiation dose low. Lopez (Lopez 2015) reported an effective dose in a large series of cases of 6 mSv.

## Clinical outcomes

Pelvic venous embolization is currently widely believed to be the best available treatment for PCS, however this is based on empirical data rather than detailed evidence from clinical trials (Borghi and Dell’Atti 2016).

In an early series, 88.9% of women reported 80% immediate relief of symptoms overall following coil embolization of the ovarian veins and contributing internal iliac tributaries. Individual symptom relief varied from 40–100% at mean follow up of 13.4 months. There were no major complications and by year 2005 centres were reporting technical success rates of 96–99% with few complications and reported rates of symptomatic relief in 75% of patients, with up to 60% experiencing complete resolution of symptoms (Drife 1993; Kuligowska et al. 2005).

A decade after the first report of OVE it was observed that it had become the mainstay of treatment, with technical success reported as 98%-100%. 70–85% patients reported immediate symptom improvement within two weeks with recurrence rates of 8% (Freedman et al. 2010; Kim et al. 2006).

In a comprehensive review of the literature in 2018, Champaniera et al. reached the following conclusion: ‘The data supporting the diagnosis and treatment of PVI in the presence of CPP are limited and of variable quality, and considerable further high-quality research is required to thoroughly address the research question’ (Champaneria et al. 2016). The group also stated ‘embolization appears to provide good to complete symptomatic relief in the majority of women’. Regarding outcomes of the 1308 patients included in the analysis 75% reported an early symptom benefit from embolization. In nine studies where pain was measured on follow up using a visual analogue scale (VAS), a statistically significant reduction was seen in all cases (Chung and Huh 2003; Venbrux 2002; Meneses et al. 2013; Kim et al. 2006; Creton et al. 2007; Richardson and Driver 2006; Gandini et al. 2008; Tropeano et al. 2008; Asciutto et al. 2009; Brown 2018). (Table2).

**Table 2:** Illustrates reported improvement in pain following ovarian vein embolization as measured by visual analogue score (VAS) in 14 published case series

Reported pain improvement following OVE for PVCS as measured by Visual Analogue Scale (VAS)						
Reference	No of women	Mean age	Initial VAS	Follow-up VAS	Length of follow up (months)	Embolic agent
Venbrux (2002)	56	32.3	7.8	2.7	12	Coils and sclerosant
Chung (2003)	52	40.1	7.8	3.2	26.6	Coils
Kim (2006)	127	34	7.8	2.9	45	Coils and sclerosant
Laborda (2013)	202	43.5	7.34	0.8	60	Coils
Hocquelet (2014)	33	41.4	7.37	1.36	26	Coils and sclerosant
Lui (2019)	12	36.5	6.7	2.7	24–36	Coils and sclerosant
De Gregori (2020)	520	43.2	7.63	0.91	59	Coils
Nasser (2014)	100	43.7	7.34	0.47	12	Coils
Senechal (2021)	327	42	6.9	2	12	Sclerosant
Gandini (2008)	38	36.9	7.8	4.2	12	Coils
Creton (2007)	24	41.5	5	1.4	36	Coils
Tropeano (2008)	22	36	8	3	12	Sclerosant
Asciutto (2009)	35	49	5.2	1.2	36	Coils
Meneses (2013)	10	38	8.2	1.36	24	Sclerosant
Mean(Range)	111 (10–520)	39 (32.3–49	7.2 (5–8.2)	2.01 (0.47–4.2)	28 (12–60)	

A further systematic review of 14 studies which examined outcomes of 994 interventions in 828 patients, 979 initial ovarian and iliac interventions (some as staged procedures) were followed by 14 repeat interventions for recurrence failure. Average follow up was 36.1 (1–288) months, clinical improvement was recorded in 68.3–100% of patients (Brown 2018).

A concern with any intervention involving the reproductive system in women is the potential impact on future fertility. A small study of 12 women reported that 66.7% (8 women) became pregnant following the procedure, 6 progressing to a live birth. They also noted that there were no differences in pre-and three months post-embolization of LH and FSH levels (*p* < 0.05) (Liu et al. 2019). Other small case series have reported pregnancies and live births following OVE (Perry 2001; Xu et al. 2016; and Yang et al. 2012). OVE has not demonstrated any hindrance or reduction in female reproductive ability.

## Current evidence and trends

A large body of literature exists related to PVCS as a cause of CPP and that the initial treatment of choice is OVE. The overwhelming consensus from the systematic reviews that have been undertaken is the need for robust multicentre randomized controlled trails (RCTs).

In 2010, the Society of Interventional Radiology (SIR) Technology Assessment Committee urged the adoption of common definitions, approaches to diagnosis, treatment, and clinical outcomes to optimize the care of patients with CPP resulting from pelvic venous insufficiency (PVI) (Black et al. 2010). They noted that although trans-catheter embolization had become an established treatment for PVCS, published outcomes were limited by non-standardized reporting, incomplete follow-up and the use of variable outcome measures.

Campbell et al. examined the opinions of vascular surgeons in the UK regarding the treatment of pelvic vein reflux (PVR) associated with varicose veins, 9% of respondents to a questionnaire did not recognize PVR as an entity and 11% do not investigate or treat it (Campbell et al. 2020). The lack of agreement on consistent terminology and the lack of prospective RCTs are cited as two reasons for these opinions.

A multidisciplinary research consensus panel identified certain areas requiring urgent research priorities in pelvic venous disorders (PeVDs) in women (Khilnani 2019). They conclude that multiple evidence gaps exist related to PeVDs with the consequence that nonvascular specialists rarely consider the diagnosis. The areas they identified are:

1. Consensus on the clinical and imaging criteria for PeVD.
2. A discriminative tool to categorize patients with PeVD.
3. QOL tools to measure the health burden in women affected by PeVD and its change after treatment.

In the USA, obtaining re-imbursement from insurance companies is challenging some considering OVE for PVI an investigative treatment, this may in part be because original studies over-emphasized the psychological component of the condition (Khilnani 2019; Bookwalter et al. 2019). They conclude that although the evidence for OVE for CPP/PVCS is strong more research is needed especially when PVCS is combined with lower extremity varicosities. Large multicentre prospective RCTs looking at pre and post embolization hormone levels, conception and pregnancy outcomes are required.

Laparoscopic trans peritoneal ovarian vein ligation is reported to be associated with symptom improvement in 75% of women. More radical surgery is only indicated in cases of unavailability or failure of less invasive techniques. OVE has reported clinical success in 70–85%, a complication rate of between 3.4–9%, however 6–31.8% of women do not get substantial or long term relief. OVE is currently believed to be the best available treatment but this is based empirical evidence rather than trials.

## Conclusion

CPP is a widespread, common and debilitating condition affecting millions of women worldwide. The aetiology is complex and multifactorial. PVCS is a common cause of CPP but is underdiagnosed. Confusion regarding the terminology used to describe the condition has added to this controversy as has the early unwarranted emphasis that is was psychosomatic in nature. The recognition that many of these women demonstrate abnormal pelvic venous vasculature was initially made either at surgery or by venography. Advances in non-invasive imaging techniques have revolutionized the diagnosis of all causes of CPP including PVCS.

OVE has superseded surgical and medical management of PVCS however the lack of robust multicentre RCTs regarding diagnosis, treatment and outcomes has hindered the complete acceptance by relevant specialists of the existence and significance of this condition. The adoption of the SVP grading system will encourage consistency of terminology, the documentation of all relevant clinical data and the standardization of reporting of outcomes. It is incumbent on all specialists undertaking OVE to engage in collaborative prospective research to enable this group of women to receive the best possible care for their condition.

## Data Availability

Not applicable.

## Abbreviations

CDI: Colour Doppler imaging
CEAP: Clinical, Etiologic, Anatomic, Physiologic
CFV: Common femoral vein
CIV: Common iliac vein
CPP: Chronic pelvic pain
CT: Computed tomography
FSH: Follicular stimulating hormone
GnRH: Gonadotrophin releasing hormone
HRT: Hormone replacement therapy
IIV: Internal iliac vein
IJV: Internal jugular vein
LH: Luteinising hormone
LOV: Left ovarian vein
MDT: Multidisciplinary team meeting
MPA: Medroxyprogesterone acetate
MRI: Magnetic resonance imaging
MRV: Magnetic resonance venography
MTS: May-Thurner syndrome
NSAIDS: Non-steroidal anti-inflammatory agents
OVE: Ovarian vein embolization
PeVDs: Pelvic venous disorders
PVCS: Pelvic venous congestion syndrome
PVI: Pelvic venous insufficiency
PVR: Pelvic vein reflux
RCT: Randomized controlled trial
ROV: Right ovarian vein
STS: Sodium tetradecyl sulphate
SVP: Symptoms-Varices-Pathophysiology
TAU: Transabdominal ultrasound
TVU: Transvaginal ultrasound
US: Ultrasound
VAS: Visual analogue scale
VV: Varicose vein
WHO: World Health Organisation

## References

[CR1] Ahlberg NE, Bartley O, Chidekel N (1966). Right and left gonadal veins: an anatomical and statistical study. Acta Radiol Diagn.

[CR2] Ahmed O, Ng J, Patel M, Ward TJ, Wang DS, Shah R, Hofmann LV (2016). Endovascular stent placement for May-Thurner syndrome in the absence of acute deep vein thrombosis. J Vasc Interv Radiol.

[CR3] Asciutto G, Mumme A, Marpe B, Köster O, Asciutto KC, Geier B (2008). MR venography in the detection of pelvic venous congestion. Eur J Vasc Endovasc Surg.

[CR4] Asciutto G, Asciutto KC, Mumme A, Geier B (2009). Pelvic venous incompetence: reflux patterns and treatment results. Eur J Vasc Endovasc Surg.

[CR5] Barge, T. F., & Uberoi, R. (2022). Symptomatic pelvic venous insufficiency: a review of the current controversies in pathophysiology, diagnosis, and management. Clin Radiol. 77:409 10.1016/j.crad.2022.01.053 35227504

[CR6] Beard, R. W., Reginald, P. W., & Wadsworth, J. (1988). Clinical features of women with chronic lower abdominal pain and pelvic congestion. Br J Obstet Gynaecol. 95(2):153–161. 10.1111/j.1471-0528.1988.tb06845.x 3349005

[CR7] Beard, R. W., Kennedy, R. G., Gangar, K. F., Stones, R. W., Rogers, V., Reginald, P. W., & Anderson, M. (1991). Bilateral oophorectomy and hysterectomy in the treatment of intractable pelvic pain associated with pelvic congestion. Br J Obstet Gynaecol. 98(10): 988–992. 10.1111/j.1471-0528.1991.tb15336.x 1751445

[CR8] Beard RW, Pearce S, Highman JH, Reginald PW (1984). Diagnosis of pelvic varicosities in women with chronic pelvic pain. Lancet.

[CR9] Beard RW, Reginald PW, Pearce S (1986). Pelvic pain in women. Br Med J (clin Res Ed).

[CR10] Brown, C. L., Rizer, M., Alexander, R., Sharpe III, E. E., & Rochon, P. J. (2018). Pelvic congestion syndrome: systematic review of treatment success. Semin Intervent Radiol. 35(1):035–040. 10.1055/s-0038-1636519 PMC5886772 29628614

[CR11] Black CM, Thorpe K, Venrbux A, Kim HS, Millward SF, Clark TW, Cardella JF (2010). Research reporting standards for endovascular treatment of pelvic venous insufficiency. J Vasc Interv Radiol.

[CR12] Bookwalter CA, VanBuren WM, Neisen MJ, Bjarnason H (2019). Imaging appearance and nonsurgical management of pelvic venous congestion syndrome. Radiographics.

[CR13] Borghi C, Dell’Atti L (2016). Pelvic congestion syndrome: the current state of the literature. Arch Gynecol Obstet.

[CR14] Campbell B, Goodyear S, Franklin I, Nyamekye I, Poskitt K (2020). Investigation and treatment of pelvic vein reflux associated with varicose veins: Current views and practice of 100 UK vascular specialists. Phlebology.

[CR15] Champaneria R, Shah L, Moss J, Gupta JK, Birch J, Middleton LJ, Daniels JP (2016). The relationship between pelvic vein incompetence and chronic pelvic pain in women: systematic reviews of diagnosis and treatment effectiveness. Health Technology Assessment (winchester, England).

[CR16] Chidekel N (1968). Female pelvic veins demonstrated by selective renal phlebography with particular reference to pelvic varicosities. Acta Radiol Diagn.

[CR17] Chung MH, Huh CY (2003). Comparison of treatments for pelvic congestion syndrome. Tohoku J Exp Med.

[CR18] Coakley FV, Varghese SL, Hricak H (1999). CT and MRI of pelvic varices in women. J Comput Assist Tomogr.

[CR19] Cordts PR, Eclavea A, Buckley PJ, DeMaioribus CA, Cockerill ML, Yeager TD (1998). Pelvic congestion syndrome: early clinical results after transcatheter ovarian vein embolization. J Vasc Surg.

[CR20] Craig O, Hobbs JT (1975). Vulval phlebography in the pelvic congestion syndrome. Clin Radiol.

[CR21] Creton D, Hennequin L, Kohler F, Allaert FA (2007). Embolization of symptomatic pelvic veins in women presenting with non-saphenous varicose veins of pelvic origin–three-year follow-up. Eur J Vasc Endovasc Surg.

[CR22] De Gregorio MA, Guirola JA, Alvarez-Arranz E, Sanchez-Ballestin M, Urbano J, Sierre S (2020). Pelvic venous disorders in women due to pelvic varices: treatment by embolization: experience in 520 patients. J Vasc Interv Radiol.

[CR23] Dick EA, Burnett C, Anstee A, Hamady M, Black D, Gedroyc WMW (2010). Time-resolved imaging of contrast kinetics three-dimensional (3D) magnetic resonance venography in patients with pelvic congestion syndrome. Br J Radiol.

[CR24] Drife JO (1993). The pelvic pain syndrome. Br J Obstet Gynaecol.

[CR25] Edwards RD, Robertson IR, MacLean AB, Hemingway AP (1993). Case report: pelvic pain syndrome–successful treatment of a case by ovarian vein embolization. Clin Radiol.

[CR26] Farquhar, C. M., Rogers, V., Franks, S., Beard, R. W., Wadsworth, J., & Pearce, S. (1989). A randomized controlled trial of medroxyprogesterone acetate and psychotherapy for the treatment of pelvic congestion. Br J Obstet Gynaecol.96(10):1153–1162. 10.1111/j.1471-0528.1989.tb03190.x 2531611

[CR27] Freedman J, Ganeshan A, Crowe PM (2010). Pelvic congestion syndrome: the role of interventional radiology in the treatment of chronic pelvic pain. Postgrad Med J.

[CR28] Gandini R, Chiocchi M, Konda D, Pampana E, Fabiano S, Simonetti G (2008). Transcatheter foam sclerotherapy of symptomatic female varicocele with sodium-tetradecyl-sulfate foam. Cardiovasc Intervent Radiol.

[CR29] Gargiulo T, Mais V, Brokaj L, Cossu E, Melis GB (2003). Bilateral laparoscopic transperitoneal ligation of ovarian veins for treatment of pelvic congestion syndrome. J Am Assoc Gynecol Laparosc.

[CR30] Gavrilov SG, Vasilyev AV, Krasavin GV, Moskalenko YP, Mishakina NY (2020). Endovascular interventions in the treatment of pelvic congestion syndrome caused by May-Thurner syndrome. J Vasc Surg Venous Lymphat Disord.

[CR31] Gloviczki, P., Comerota, A. J., Dalsing, M. C., Eklof, B. G., Gillespie, D. L., Gloviczki, M. L., ... & Wakefield, T. W. (2011). The care of patients with varicose veins and associated chronic venous diseases: clinical practice guidelines of the Society for Vascular Surgery and the American Venous Forum. J Vasc Surg. 53(5), 2S-48S. 10.1016/j.jvs.2011.01.079 21536172

[CR32] Gong, M., He, X., Zhao, B., Kong, J., Gu, J., & Su, H. (2021). Ovarian Vein Embolization with N-butyl-2 Cyanoacrylate Glubran-2® for the Treatment of Pelvic Venous Disorder. Front Surg. 8: 657-663 https://www.frontiersin.org/articles/10.3389/fsurg.2021.760600/full. 10.3389/fsurg.2021.760600 PMC8716375 34977140

[CR33] Guilhem, D. P., & Baux, D. R. (1954).La Phlébographie pelvienne par voies veineuse, osseuse et utérine: application à l'étude des phlébites et des cancers, par P. Guilhem... et R. Baux... Préface du Prof.[Joseph] Ducuing. Masson.

[CR34] Gynaecological Laparoscopy Report of the Working Party of the Confidential Enquiry into Gynaecological Laparoscopy (1978). Chamberlain, and IC Brown, eds., RCOG London 10.1111/j.1471-0528.1978.tb14904.x 148903

[CR35] Hobbs JT (1976). The pelvic congestion syndrome. Practitioner.

[CR36] Hocquelet A, Le Bras Y, Balian E, Bouzgarrou M, Meyer M, Rigou G, Grenier N (2014). Evaluation of the efficacy of endovascular treatment of pelvic congestion syndrome. Diagn Interv Imaging.

[CR37] Hodgson TJ, Reed MW, Peck RJ, Hemingway AP (1991). Case report: the ultrasound and Doppler appearances of pelvic varices. Clin Radiol.

[CR38] Ignacio, E. A., Dua, R., Sarin, S., Harper, A. S., Yim, D., Mathur, V., & Venbrux, A. C. (2008). Pelvic congestion syndrome: diagnosis and treatment. Semin Intervent Radiol. 25(4):361–368. 10.1055/s-0028-1102998 PMC3036528 21326577

[CR39] Jacobs JB (1969). Selective gonadal venography. Radiology.

[CR40] Kauppila A, Järvinen PA, Vuorinen P (1971). Improved visualization in uterine phlebography. Br J Radiol.

[CR41] Kennedy A, Hemingway A (1990). Radiology of ovarian varices. Br J Hosp Med.

[CR42] Khilnani, N. M., Meissner, M. H., Learman, L. A., Gibson, K. D., Daniels, J. P., Winokur, R. S., ... & Rosenblatt, M. (2019). Research priorities in pelvic venous disorders in women: recommendations from a multidisciplinary research consensus panel. J Vasc Intervent Radiol. 30(6), 781–789. 10.1016/j.jvir.2018.10.008 30857986

[CR43] Kim HS, Malhotra AD, Rowe PC, Lee JM, Venbrux AC (2006). Embolotherapy for pelvic congestion syndrome: long-term results. J Vasc Interv Radiol.

[CR44] Knuttinen MG, Xie K, Jani A, Palumbo A, Carrillo T, Mar W (2015). Pelvic venous insufficiency: imaging diagnosis, treatment approaches, and therapeutic issues. Am J Roentgenol.

[CR45] Kuligowska E, Deeds L, Lu K (2005). Pelvic pain: overlooked and underdiagnosed gynecologic conditions. Radiographics.

[CR46] Laborda A, Medrano J, de Blas I, Urtiaga I, Carnevale FC, de Gregorio MA (2013). Endovascular treatment of pelvic congestion syndrome: visual analog scale (VAS) long-term follow-up clinical evaluation in 202 patients. Cardiovasc Intervent Radiol.

[CR47] Lefevre H (1964). Broad ligament varicocele. Acta Obstet Gynecol Scand.

[CR48] Lemasle P, Greiner M (2017). Duplex ultrasound investigation in pelvic congestion syndrome: technique and results. Phlebolymphology.

[CR49] Liu J, Han L, Han X (2019). The effect of a subsequent pregnancy after ovarian vein embolization in patients with infertility caused by pelvic congestion syndrome. Acad Radiol.

[CR50] Lopez AJ (2015). Female pelvic vein embolization: indications, techniques, and outcomes. Cardiovasc Intervent Radiol.

[CR51] Lurie F, Passman M, Meisner M, Dalsing M, Masuda E, Welch H, Wakefield T (2020). The 2020 update of the CEAP classification system and reporting standards. J Vasc Surg Venous Lymphat Disord.

[CR52] Mathias SD, Kuppermann M, Liberman RF, Lipschutz RC, Steege JF (1996). Chronic pelvic pain: prevalence, health-related quality of life, and economic correlates. Obstet Gynecol.

[CR53] Meissner MH, Khilnani NM, Labropoulos N, Gasparis AP, Gibson K, Greiner M, Rosenblatt M (2021). The symptoms-varices-pathophysiology classification of pelvic venous disorders: a report of the American Vein & Lymphatic Society international working group on pelvic venous disorders. Phlebology.

[CR54] Meneses L, Fava M, Diaz P, Andía M, Tejos C, Irarrazabal P, Uribe S (2013). Embolization of incompetent pelvic veins for the treatment of recurrent varicose veins in lower limbs and pelvic congestion syndrome. Cardiovasc Intervent Radiol.

[CR55] Nasser F, Cavalcante RN, Affonso BB, Messina ML, Carnevale FC, de Gregorio MA (2014). Safety, efficacy, and prognostic factors in endovascular treatment of pelvic congestion syndrome. Int J Gynecol Obstet.

[CR56] Osman AM, Mordi A, Khattab R (2021). Female pelvic congestion syndrome: how can CT and MRI help in the management decision?. Br J Radiol.

[CR57] Pain, C. P. (2020). ACOG Practice Bulletin Number 218 Obstet Gynecol 3:e98-e109. 10.1097/AOG.0000000000003716 32080051

[CR58] Park SJ, Lim JW, Ko YT, Lee DH, Yoon Y, Oh JH, Huh CY (2004). Diagnosis of pelvic congestion syndrome using transabdominal and transvaginal sonography. Am J Roentgenol.

[CR59] Perry, C. P. (2001). Current concepts of pelvic congestion and chronic pelvic pain. JSLS. 5(2):105. PMC3015423 11394421

[CR60] Phillips D, Deipolyi AR, Hesketh RL, Midia M, Oklu R (2014). Pelvic congestion syndrome: etiology of pain, diagnosis, and clinical management. J Vasc Interv Radiol.

[CR61] Richardson GD, Driver B (2006). Ovarian vein ablation: coils or surgery?. Phlebology.

[CR62] Richet MA (1857) Traité pratique d’anatomie médico-chirurgicaleE Chaamerot. Libraairee Editeur, Paris

[CR63] Senechal, Q., Echegut, P., Bravetti, M., Florin, M., Jarboui, L., Bouaboua, M & Pessis, E. (2021). Endovascular treatment of pelvic congestion syndrome: visual analog scale follow-up. Front Cardiovasc Med, 1518. 10.3389/fcvm.2021.751178 PMC8635860 34869656

[CR64] Scott L, Cullen J (2021). Incidental finding of an asymptomatic migrated coil to the right ventricle following pelvic vein embolization. Vascular & Endovascular Review.

[CR65] Silverberg PW, Slowinski EJ, Melnick GS (1973). Pelvic venography. Radiology.

[CR66] Stones RW, Rae T, Rogers V, Fry R, Beard RW (1990). Pelvic congestion in women: evaluation with transvaginal ultrasound and observation of venous pharmacology. Br J Radiol.

[CR67] Stovall TG, Ling FW, Crawford DA (1990). Hysterectomy for chronic pelvic pain of presumed uterine etiology. Obstet Gynecol.

[CR68] Szaflarski D, Sosner E, French TD, Sayegh S, Lamba R, Katz DS, Hoffmann JC (2019). Evaluating the frequency and severity of ovarian venous congestion on adult computed tomography. Abdominal Radiology.

[CR69] Taylor HC (1949). Vascular congestion and hyperemia: their effect on structure and function in the female reproductive system. Am J Obstet Gynecol.

[CR70] Tessari L, Cavezzi A, Frullini A (2001). Preliminary experience with a new sclerosing foam in the treatment of varicose veins. Dermatol Surg.

[CR71] Thomas ML, Fletcher EWL, Andress MR, Cockett FB (1967). The venous connections of vulval varices. Clin Radiol.

[CR72] Thorne C, Stuckey B (2008). CASE REPORT: Pelvic Congestion Syndrome Presenting as Persistent Genital Arousal: A Case Report. J Sex Med.

[CR73] Tropeano G, Di Stasi C, Amoroso S, Cina A, Scambia G (2008). Ovarian vein incompetence: a potential cause of chronic pelvic pain in women. Eur J Obstetr Gynecol Reprod Biol.

[CR74] Venbrux AC, Chang AH, Kim HS, Montague BJ, Hebert JB, Arepally A, Robinson JC (2002). Pelvic congestion syndrome (pelvic venous incompetence): impact of ovarian and internal iliac vein embolotherapy on menstrual cycle and chronic pelvic pain. J Vasc Int Radiol.

[CR75] Venbrux, A.C., Sharma, G.K., Jackson, E.T., Harper, A.P., Hover, L. (2012). Pelvic Varices Embolization. In: Ignacio, E., Venbrux, A. (eds) Women’s Health in Interventional Radiology. Springer, New York, NY. pp 37-59 https://doi.org/10.1007/978-1-4419-5876-1_2

[CR76] WHO Surgical Safety Checklist. https://www.who.int/teams/integrated-health-services/patient-safety/research/safe-surgery/tooland-resources. Accessed 3 Apr 2023

[CR77] Xu J, Wang YF, Chen AW, Wang T, Liu SH (2016). A modified Tessari method for producing more foam. Springerplus.

[CR78] Yang DM, Kim HC, Nam DH, Jahng GH, Huh CY, Lim JW (2012). Time-resolved MR angiography for detecting and grading ovarian venous reflux: comparison with conventional venography. Br J Radiol.

[CR79] Zurcher, K. S., Staack, S. O., Spencer, E. B., Liska, A., Alzubaidi, S. J., Patel, I. J., ... & Knuttinen, M. G. (2022). Venous Anatomy and Collateral Pathways of the Pelvis: An Angiographic Review. RadioGraphics. 42(5):1532–1545. 10.1148/rg.220012 35867595

